# Prognostic implications of thyroid hormones in acute aortic dissection: mediating roles of renal function and coagulation

**DOI:** 10.3389/fendo.2024.1387845

**Published:** 2024-08-02

**Authors:** Xuejun Shen, Shiwan Wu, Jingyi Yan, Hongle Yan, Shuyi Zhou, Huozhen Weng, Shengli Yang, Weiping Li

**Affiliations:** ^1^ Department of Cardiology, First Affiliated Hospital of Shantou University Medical College, Shantou, Guangdong, China; ^2^ Clinical Research Center, First Affiliated Hospital of Shantou University Medical College, Shantou, Guangdong, China; ^3^ Shantou University Medical College, Shantou, Guangdong, China

**Keywords:** acute aortic dissection (AAD), thyroid hormones, coagulation, major adverse cardiovascular events (MACEs), causal mediation analysis

## Abstract

**Background:**

Thyroid hormones significantly influence cardiovascular pathophysiology, yet their prognostic role in acute aortic dissection (AAD) remains inadequately explored. This study assesses the prognostic value of thyroid hormone levels in AAD, focusing on the mediating roles of renal function and coagulation.

**Methods:**

We included 964 AAD patients in this retrospective cohort study. Utilizing logistic regression, restricted cubic splines, and causal mediation analysis, we investigated the association between thyroid hormones and in-hospital mortality and major adverse cardiovascular events (MACEs).

**Results:**

In AAD patients overall, an increase of one standard deviation in FT4 levels was associated with a 31.9% increased risk of MACEs (OR 1.319; 95% CI 1.098–1.584) and a 36.1% increase in in-hospital mortality (OR 1.361; 95% CI 1.095–1.690). Conversely, a higher FT3/FT4 ratio was correlated with a 20.2% reduction in risk of MACEs (OR 0.798; 95% CI 0.637–0.999). This correlation was statistically significant predominantly in Type A AAD, while it did not hold statistical significance in Type B AAD. Key renal and coagulation biomarkers, including blood urea nitrogen, creatinine, cystatin C, prothrombin time ratio, prothrombin time, and prothrombin time international normalized ratio, were identified as significant mediators in the interplay between thyroid hormones and MACEs. The FT3/FT4 ratio exerted its prognostic influence primarily through the mediation of renal functions and coagulation, while FT4 levels predominantly impacted outcomes via a partial mediation effect on coagulation.

**Conclusion:**

FT4 levels and the FT3/FT4 ratio are crucial prognostic biomarkers in AAD patients. Renal function and coagulation mediate the association between the thyroid hormones and MACEs.

## 1 Introduction

Acute aortic dissection (AAD) is a life-threatening cardiovascular condition with a prognosis influenced by various factors, including the patient’s overall health status, disease severity, and chosen treatment modalities. Despite advancements in understanding these contributory factors, mortality rates in AAD patients remain alarmingly high (1, 2). Therefore, identifying novel prognostic indicators and comprehensively understanding their roles in adverse outcomes are crucial.

Thyroid hormones are recognized as metabolic regulators with intricate interactions within the cardiovascular system. These interactions manifest through various mechanisms, including dyslipidemia, endothelial dysfunction, alterations in blood pressure, and direct effects on myocardial tissue (3, 4). Evidence has shown that thyroid hormones are associated with adverse prognostic outcomes in cardiovascular patients (5–9). Among the thyroid hormones, free triiodothyronine (FT3) and free thyroxine (FT4) are pivotal in influencing both physiological and pathological cardiovascular processes. The FT3/FT4 ratio, a marker for thyroid hormone metabolism and conversion from T4 to T3, has demonstrated prognostic utility in various cardiovascular diseases (10–12). Previous small-scale studies have explored the effects of T3 or T4 on postoperative acute kidney injury (13) and postoperative delirium (14), as well as the relationship between high-thyroid stimulating hormone (TSH) subclinical hypothyroidism and postoperative mortality in AAD patients (15). However, a comprehensive examination of the prognostic significance of the FT3/FT4 ratio in AAD patients remains limited.

Renal function and coagulation are critical determinants of cardiovascular disease prognosis. Impaired renal function is associated with increased mortality and adverse cardiovascular events (16, 17), while coagulation abnormalities can lead to thromboembolic events and worsen cardiovascular outcomes (18–20). Given the known interactions between thyroid function, renal function (21–24), and coagulation (25–27), we hypothesize that thyroid function may influence AAD prognosis through its effects on renal function and coagulation, which serve as mediating factors in this relationship.

The primary objective of this study is to elucidate the relationship between thyroid hormones and adverse outcomes in AAD patients, while also assessing the mediating roles of renal function and coagulation, to offer insight into the potential biological mechanisms of AAD.

## 2 Method

### 2.1 Study population

**Figure 1:**
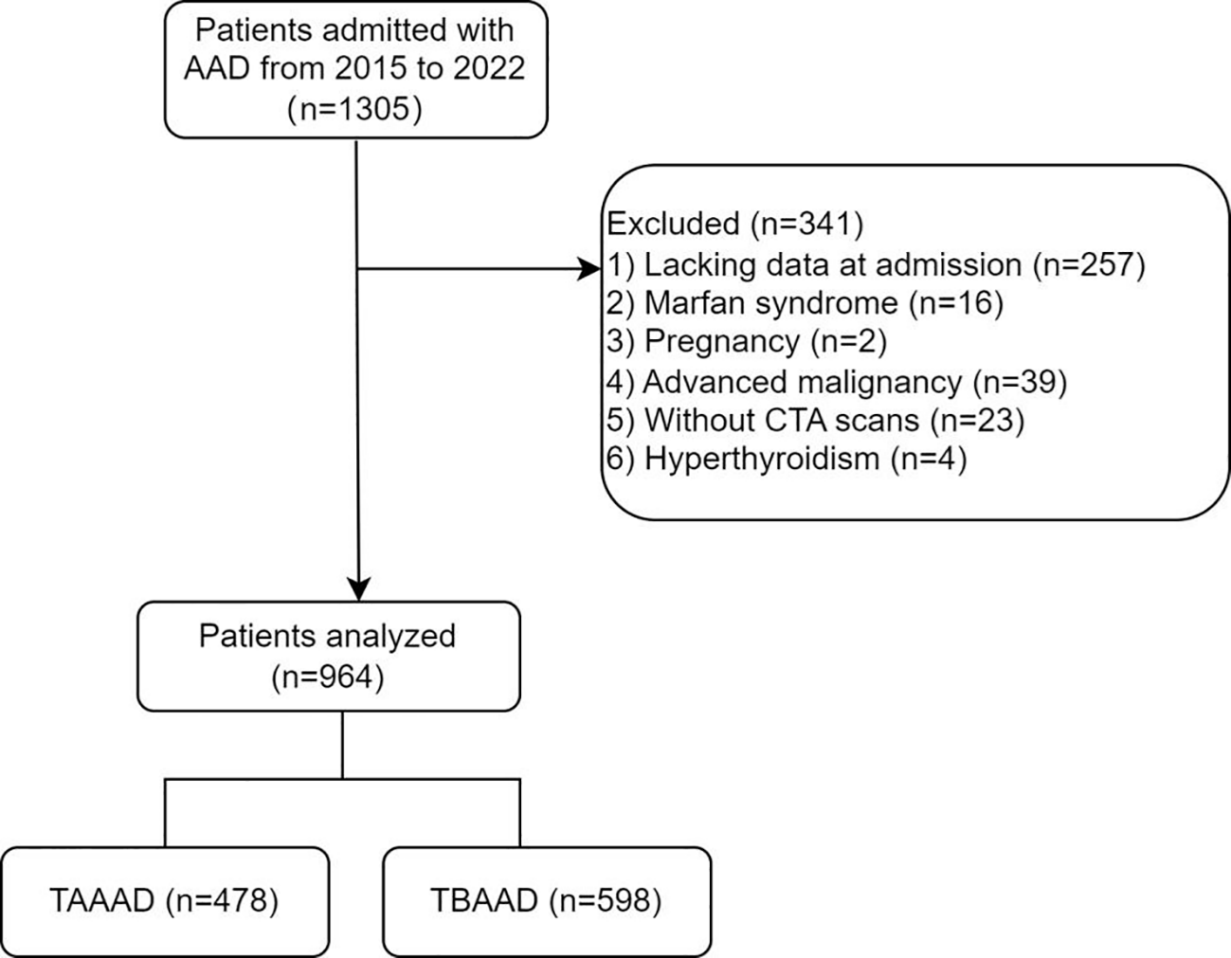
Flow diagram of patient selection.

The study cohort comprised 1,305 patients diagnosed with AAD at the First Affiliated Hospital of Shantou University Medical College between 2015 and 2022, and only the initial admission of each patient was considered. Serum FT3 and FT4 levels were evaluated upon admission. Exclusion criteria encompassed missing FT3, FT4, or computed tomography angiography data, prior hyperthyroidism, pregnancy, Marfan syndrome, concurrent malignant tumor, or being underage. Following these criteria, a total of 964 patients were enrolled for this investigation (**Figure 1**). This retrospective study conforms to the stipulations of the Declaration of Helsinki, and received approval from the Ethics Committee of the First Affiliated Hospital of Shantou University Medical College.

### 2.2 Data collection and definitions

Data were retrospectively extracted from electronic medical records including demographic variables (e.g., age and gender) and pertinent clinical history variables, including hypertension, diabetes mellitus, atrial fibrillation, coronary artery disease, and smoking status. Laboratory tests included thyroid function tests (FT3, FT4, and TSH); renal function tests, such as blood urea nitrogen (BUN), creatinine (Cr), and cystatin C (CysC); and a lipid profile encompassing low-density lipoprotein cholesterol (LDL-C), high-density lipoprotein cholesterol (HDL-C), total cholesterol (TC), and triglycerides (TRIG). Additionally, serum albumin, coagulation markers, and a complete blood count were assessed. We also calculated estimated glomerular filtration rate (eGFR) using the Chronic Kidney Disease Epidemiology Collaboration (CKD-EPI) equation (28, 29).

Due to the critical condition of many patients with AAD, anthropometric measurements, such as height and weight, were often unattainable. Consequently, owing to the elevated proportion of missing data, body mass index (BMI) was not included as a variable. Instead, the controlling nutritional status (CONUT) score, a validated instrument for nutritional risk assessment, was utilized as a covariate. The CONUT score is a validated tool for screening and identifying malnutrition in hospitalized patients, calculated based on serum albumin levels, total cholesterol, and total lymphocyte count (30, 31).

Detailed records were maintained for all therapeutic interventions, including surgical procedures, thoracic endovascular aortic repair (TEVAR), and pharmacological treatments. The pharmacological regimens included β-adrenergic blockers, angiotensin-converting enzyme inhibitors (ACEIs), calcium channel blockers (CCBs), and statins. Based on computed tomography angiography results, patients were categorized as either Type A acute aortic dissection (TAAAD) or Type B acute aortic dissection (TBAAD).

### 2.3 Endpoints

The primary endpoints of this study were in-hospital mortality and major adverse cardiovascular events (MACEs). MACEs were defined as a composite of acute myocardial infarction, repeat revascularization, heart failure, stroke, vascular reconstruction (primarily coronary artery bypass grafting and percutaneous coronary intervention), and all-cause mortality.

### 2.4 Statistical analysis

For continuous variables, those with normal or near-normal distributions are represented as mean (standard deviation, SD), while those with skewed distributions are described using the median (interquartile range, IQR). Categorical variables are denoted as numbers (percentages, %). The chi-squared test was utilized for categorical variables, ANOVA for normally distributed continuous variables, and the Kruskal-Wallis test for skewed continuous variables.

Non-linear associations between FT3, FT4, or the FT3/FT4 ratio and in-hospital mortality, as well as MACEs, were assessed using restricted cubic splines (RCS). Furthermore, we standardized (Z-score) the FT3, FT4, and FT3/FT4 ratio, then included them in the univariate and multivariate logistic analyses to investigate the odds ratios (OR) and 95% confidence intervals (CI) for each SD increase. Model 1 adjusted for age, gender, AAD type, hypertension, diabetes, surgical intervention, and TEVAR. Model 2 incorporated additional variables such as smoking, use of CCBs, and β-blockers, and Model 3 further adjusted for TSH, CKMB, WBC, AST, and CONUT score. Subgroup analyses were stratified according to the type of aortic dissection (TAAAD or TBAAD). Additionally, causal mediation analysis was used to probe the mediating effects of BUN, Cr, CysC, eGFR, prothrombin time (PT), prothrombin time ratio (PTR), and prothrombin time international normalized ratio (PTINR) on the FT3/FT4 ratio-MACEs association, as well as their mediating effects on the FT4-MACEs association, employing the ‘mediation’ and ‘bruceR’ packages in R.

All analyses were performed using R (version 4.3.0), and a p-value of <0.05 was considered statistically significant.

## 3 Results

### 3.1 Participant characteristics

Patient demographics and clinical characteristics are listed in **Table 1**. Of the 964 patients with acute aortic dissection (AAD) enrolled, 478 were diagnosed with TAAAD and 486 with TBAAD. The average age of the cohort was 58 years, with a noticeable male preponderance. When examining medical histories, hypertension was prominently present in 76% of the cohort. There were no statistically significant differences between the TAAAD and TBAAD subgroups in relation to hypertension, diabetes mellitus, atrial fibrillation, or coronary artery disease. However, therapeutic interventions showed a significant disparity between the TBAAD and TAAAD subgroups, particularly regarding the incidence of surgical or endovascular stent-graft procedures. Medication use was also markedly elevated in the TBAAD subgroup. Distinct differences between the subgroups emerged in various laboratory indices. A significant difference in smoking prevalence was observed between the TBAAD and TAAAD groups, as well.

**Table 1:** Patient demographics and clinical characteristics.

Characteristic	ADD type			*P*-value
	Overall, N = 964	A, N = 478	B, N = 486	
Demographics				
Age (years)	58.0 (50.00, 67.00)	58.0 (50.00, 66.00)	58.0 (49.25, 68.75)	0.312
Gender (male) n (%)	755 (78%)	346 (72%)	409 (84%)	<0.001
Smoker n (%)	587 (61%)	271 (57%)	316 (65%)	0.008
Medical history, n (%)				
Hypertension	737 (76%)	358 (75%)	379 (78%)	0.259
Diabetes	71 (7%)	33 (7%)	38 (8%)	0.587
Atrial fibrillation	23 (2%)	15 (3%)	8 (2%)	0.129
CHD	40 (4%)	15 (3%)	25 (5%)	0.118
Treatment, n (%)				
Surgery	313 (32%)	297 (62%)	16 (3%)	<0.001
TEVAR	239 (25%)	9 (2%)	230 (47%)	<0.001
ACEI	371 (38%)	127 (27%)	244 (50%)	<0.001
β-Blocker	832 (86%)	374 (78%)	458 (94%)	<0.001
CCB	754 (78%)	314 (66%)	440 (91%)	<0.001
α-Blocker	393 (41%)	130 (27%)	263 (54%)	<0.001
ARB	247 (26%)	74 (15%)	173 (36%)	<0.001
Statin	431 (45%)	166 (35%)	265 (55%)	<0.001
Diuretic	358 (37%)	151 (32%)	207 (43%)	<0.001
Vital Signs				
SBP (mmHg)	155.9 (34.62)	147.0 (33.75)	164.6 (33.25)	<0.001
DBP (mmHg)	88.0 (74.75, 103.00)	82.0 (67.00, 97.00)	94.0 (81.00, 107.00)	<0.001
Heart rate (bpm)	78.0 (68.00, 90.00)	78.0 (66.25, 90.00)	78.0 (69.00, 89.75)	0.480
Temperature (°C)	36.5 (36.20, 36.70)	36.5 (36.20, 36.70)	36.5 (36.30, 36.70)	0.152
Laboratory data				
CK (U/L)	94.9 (58.49, 170.00)	107.8 (65.61, 200.74)	84.5 (55.00, 142.04)	<0.001
CKMB (U/L)	13.0 (10.06, 19.00)	14.0 (10.85, 23.14)	12.1 (10.00, 16.00)	<0.001
AST (U/L)	22.0 (17.00, 35.67)	26.0 (18.97, 57.22)	20.0 (16.00, 26.00)	<0.001
ALT (U/L)	19.0 (13.00, 32.35)	22.0 (14.10, 40.67)	17.0 (12.00, 26.00)	<0.001
TP (g/L)	64.6 (60.78, 68.11)	64.1 (60.13, 67.50)	65.2 (61.70, 68.99)	<0.001
ALB (g/L)	36.5 (33.80, 39.02)	36.4 (33.64, 38.63)	36.7 (33.89, 39.24)	0.190
FBG (mmol/L)	6.9 (5.88, 8.35)	7.2 (6.17, 8.90)	6.6 (5.59, 7.58)	<0.001
BUN (mmol/L)	7.2 (5.71, 9.72)	7.8 (6.23, 10.35)	6.7 (5.18, 9.00)	<0.001
Cr (μmol/L)	112.0 (89.57, 146.05)	117.8 (94.73, 155.75)	107.0 (86.42, 136.71)	<0.001
CysC (mg/L)	1.0 (0.81, 1.45)	1.0 (0.81, 1.51)	1.0 (0.82, 1.34)	0.201
eGFR(mL/min/1.73 m^2^)	58.5 (43.12, 77.21)	55.3 (38.60, 70.40)	62.8 (47.00, 80.70)	<0.001
UA (μmol/L)	422.0 (333.00, 505.80)	445.7 (348.86, 520.60)	408.9 (323.73, 496.00)	0.002
Chol (mmol/L)	4.5 (3.89, 5.12)	4.5 (3.82, 5.07)	4.5 (3.91, 5.19)	0.078
TRIG (mmol/L)	1.1 (0.82, 1.53)	1.1 (0.83, 1.43)	1.1 (0.82, 1.60)	0.133
HDL (mmol/L)	1.1 (0.95, 1.31)	1.2 (0.97, 1.33)	1.1 (0.92, 1.28)	0.005
LDL (mmol/L)	2.8 (2.38, 3.35)	2.8 (2.34, 3.29)	2.9 (2.40, 3.40)	0.039
FT3 (pmol/L)	4.2 (3.76, 4.69)	4.1 (3.68, 4.62)	4.3 (3.86, 4.77)	<0.001
FT4 (pmol/L)	11.7 (10.49, 13.07)	11.9 (10.72, 13.21)	11.6 (10.33, 12.94)	0.013
TSH (mIU/L)	0.7 (0.41, 1.14)	0.7 (0.40, 1.15)	0.7 (0.42, 1.13)	0.518
FT3/FT4	0.4 (0.31, 0.43)	0.3 (0.29, 0.41)	0.4 (0.32, 0.44)	<0.001
WBC (10^9/L)	11.7 (9.16, 14.39)	12.6 (10.08, 15.42)	10.7 (8.42, 13.13)	<0.001
LY (10^9/L)	1.1 (0.76, 1.49)	1.0 (0.71, 1.39)	1.1 (0.81, 1.55)	0.005
PT (seconds)	11.5 (10.80, 12.30)	11.8 (11.00, 12.70)	11.2 (10.60, 12.00)	<0.001
PTR	1.0 (0.94, 1.07)	1.0 (0.96, 1.11)	1.0 (0.93, 1.04)	<0.001
Fib (g/L)	2.9 (2.13, 3.90)	2.6 (1.83, 3.58)	3.2 (2.45, 4.21)	<0.001
PTINR	1.0 (0.94, 1.07)	1.0 (0.95, 1.12)	1.0 (0.93, 1.05)	<0.001

ALB, albumin; ALT, alanine aminotransferase; AST, aspartate aminotransferase; BUN, blood urea nitrogen; Chol, cholesterol; CK, creatine kinase; CKMB, creatine kinase-MB; Cr, creatinine; CysC, cystatin C; eGFR: estimated glomerular filtration rate; FBG, fasting blood glucose; Fib, fibrinogen; FT3, free triiodothyronine; FT3/FT4, ratio of free triiodothyronine to free thyroxine; FT4, free thyroxine; HDL, high-density lipoprotein; LDL, low-density lipoprotein; LY, lymphocyte count; PT, prothrombin time; PTINR, prothrombin time international normalized ratio; PTR, prothrombin time ratio; TP, total protein; TRIG, triglycerides; TSH, thyroid-stimulating hormone; UA, uric acid; WBC, white blood cell count.

### 3.2 Thyroid hormone levels, MACEs and in-hospital mortality in AAD patients

**Figure 2:**
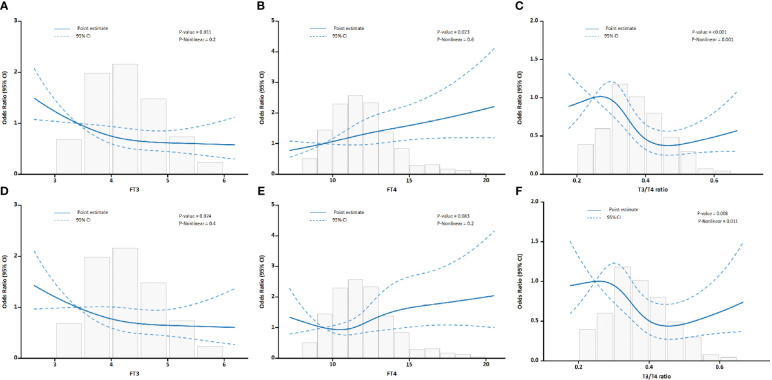
Association between FT3, FT4, and the FT3/FT4 ratio with outcomes in all AAD patients. **(A)** Association of FT3 with MACEs. **(B)** Association of FT4 with MACEs. **(C)** Association of the FT3/FT4 ratio with MACEs. **(D)** Association of FT3 with in-hospital mortality. **(E)** Association of FT4 with in-hospital mortality. **(F)** Association of the FT3/FT4 ratio with in-hospital mortality.

The relationship between thyroid hormone levels and the incidence of MACEs as well as in-hospital mortality in patients with AAD was assessed. Initially, unadjusted RCS were employed for visual representation of these associations. As illustrated in **Figure 2**, a progressive escalation in the incidence rates of both MACEs and in-hospital mortality was discernible with incremental elevations in serum FT4 concentrations. In contrast, higher serum FT3 levels showed an inverse association. Notably, the FT3/FT4 ratio displayed an S-shaped non-linear correlation with adverse prognostic outcomes in AAD patients. The curve was characterized by a more gradual change at its extremities, contrasting with the significant decline observed in the mid-range, especially between the 0.25 and 0.45 values.

After adjusting for confounding variables, as shown in **Table 2**, each SD increase in the FT3/FT4 ratio corresponded to a 20.2% decrease in the risk of MACEs (OR, 0.798; 95% CI, 0.637–0.999). Conversely, each SD increase in FT4 level was associated with a 31.9% increased risk for MACEs (OR 1.319; 95% CI, 1.098–1.584). The association between FT3 and MACEs became insignificant after further multivariable adjustments. This implies that the FT3/FT4 ratio may be a more accurate representation of thyroid hormone metabolic variations linked to MACEs in AAD patients than FT3 or FT4 alone. For in-hospital mortality, both FT3 and the FT3/FT4 ratio showed inverse relationships in univariate analyses. However, these associations were not significant in multivariable models. FT4 consistently correlated positively with in-hospital mortality after adjusting for all confounding variables.

**Table 2:** Odds ratios for MACEs and in-hospital mortality associated with FT3, FT4, and FT3/FT4 levels (per SD increase) in AAD patients.

	FT3		FT4		FT3/FT4	
	OR(95% CI)	*P-*value	OR(95% CI)	*P-*value	OR(95% CI)	*P-*value
MACEs						
Unadjusted	0.814 (0.700–0.948)	0.008	1.216 (1.057–1.400)	0.006	0.756 (0.642–0.889)	<0.001
Model 1	0.939 (0.796–1.109)	0.46	1.186 (1.021–1.387)	0.026	0.836 (0.709–0.986)	0.034
Model 2	0.952 (0.806–1.124)	0.564	1.191 (1.022–1.387)	0.025	0.844 (0.716–0.995)	0.044
Model 3	1.027 (0.854–1.236)	0.775	1.319 (1.098–1.584)	0.003	0.798 (0.637–0.999)	0.050
In-hospital mortality						
Unadjusted	0.828 (0.689–0.995)	0.044	1.181 (1.004–1.389)	0.045	0.822 (0.678–0.997)	0.047
Model 1	0.968 (0.785–1.193)	0.758	1.164 (0.976–1.389)	0.092	0.903 (0.742–1.099)	0.308
Model 2	0.981 (0.795–1.209)	0.854	1.158 (0.964–1.390)	0.117	0.921 (0.758–1.119)	0.409
Model 3	1.025 (0.814–1.291)	0.830	1.361 (1.095–1.690)	0.005	0.815 (0.629–1.056)	0.121

Model adjustments:

Model 1: adjusted for age, gender, AAD type, hypertension, diabetes, surgical intervention, and TEVAR.

Model 2: further adjusted for smoking status and usage of CCBs and β-blockers.

Model 3: further adjusted for CONUT score, WBC, AST, CKMB, and TSH.

### 3.3 Interplay between thyroid hormone levels and subtypes of AAD: a stratified analysis based on Stanford classification

Patients were categorized into Stanford Type A and Type B subgroups, with separate analyses conducted for each. The pertinent statistical outcomes are presented in **Tables 3** and **4**.

**Table 3:** Odds ratios for MACEs and in-hospital mortality associated with FT3, FT4, and FT3/FT4 levels (per SD increase) in TAAAD patients.

	FT3		FT4		FT3/FT4 ratio	
	OR(95% CI)	*P*-value	OR(95% CI)	*P*-value	OR(95% CI)	*P*-value
MACEs						
Unadjusted	0.848 (0.696–1.033)	0.101	1.202 (0.995–1.451)	0.056	0.750 (0.605–0.930)	0.009
Model 1	0.924 (0.741–1.151)	0.481	1.287 (1.050–1.578)	0.015	0.745 (0.594–0.933)	0.01
Model 2	0.948 (0.759–1.183)	0.508	1.277 (1.036–1.574)	0.022	0.773 (0.615–0.971)	0.027
Model 3	0.989 (0.765–1.279)	0.735	1.457 (1.115–1.904)	0.006	0.687 (0.512–0.921)	0.012
In-hospital mortality						
Unadjusted	0.817 (0.654–1.021)	0.076	1.166 (0.954–1.425)	0.134	0.791 (0.624–1.002)	0.052
Model 1	0.870 (0.668–1.134)	0.303	1.293 (1.035–1.615)	0.024	0.752 (0.580–0.975)	0.031
Model 2	0.891 (0.684–1.162)	0.395	1.274 (1.007–1.611)	0.043	0.791 (0.607–1.030)	0.082
Model 3	0.940 (0.692–1.276)	0.690	1.521 (1.142–2.026)	0.004	0.730 (0.519–1.028)	0.072

Model Adjustments:

Model 1: Adjusted for age, gender, hypertension, diabetes, and surgical intervention.

Model 2: Further adjusted for smoking status and usage of CCB and β-blockers.

Model 3: Further adjusted for CONUT score, WBC, AST, CKMB, and TSH.

**Table 4:** Odds ratios for MACEs and in-hospital mortality associated with FT3, FT4, and FT3/FT4 levels (per SD increase) in TBAAD patients.

	FT3		FT4		FT3/FT4 ratio	
	OR(95% CI)	*P*-value	OR(95% CI)	*P*-value	OR(95% CI)	*P*-value
MACEs						
Unadjusted	0.789 (0.558–1.116)	0.181	1.056 (0.952–1.173)	0.303	0.244 (0.02–3.006)	0.271
Model 1	0.933 (0.651–1.337)	0.705	1.019 (0.916–1.133)	0.733	0.825 (0.071–9.59)	0.878
Model 2	0.93 (0.649–1.334)	0.694	1.028 (0.924–1.144)	0.609	0.63 (0.053–7.447)	0.714
Model 3	1.119 (0.758–1.651)	0.572	1.082 (0.963–1.216)	0.185	0.207 (0.009–4.853)	0.328
In-hospital mortality						
Unadjusted	1.124 (0.692–1.825)	0.637	0.979 (0.837–1.145)	0.794	5.422 (0.229–128.562)	0.295
Model 1	1.325 (0.798–2.200)	0.277	0.956 (0.818–1.117)	0.569	12.104 (0.541–270.868)	0.116
Model 2	1.330 (0.800–2.213)	0.272	0.956 (0.817–1.118)	0.569	13.092 (0.536–319.662)	0.115
Model 3	1.416 (0.848–2.364)	0.184	1.034 (0.870–1.230)	0.703	2.216 (0.03–164.595)	0.717

Model Adjustments:

Model 1: Adjusted for age, gender, hypertension, diabetes, and TEVAR.

Model 2: Further adjusted for smoking status and usage of CCB and β-blockers.

Model 3: Further adjusted for CONUT score, WBC, AST, CKMB, and TSH.

In the analysis of TAAAD patients, our data demonstrated a consistent association, mirroring the observations made in the overall AAD cohort. Specifically, with each increase of one SD in the FT3/FT4 ratio, we identified a 31.3% reduction in risk of MACEs (OR: 0.687; 95% CI: 0.512–0.921). Conversely, an increase in FT4 level was significantly correlated with an elevated risk of adverse outcomes. Each SD increment in FT4 was associated with a 45.7% increase in risk of MACEs (OR: 1.457; 95% CI: 1.115–1.904) and a 52.1% increase in in-hospital mortality risk (OR: 1.521; 95% CI: 1.142–2.026). However, these observed relationships were conspicuously absent in the TBAAD cohort.

This detailed observation underscores the complex relationship between thyroid hormone levels and acute aortic dissection, and suggests that the nature of this relationship may vary among different AAD subtypes.

### 3.4 Linear relationship between FT4, FT3/FT4 ratio and MACEs in TAAAD patients

**Figure 3:**
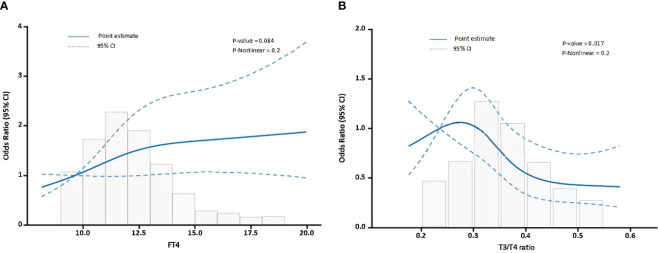
The relationship between FT4, FT3/FT4 ratio and MACEs in TAAAD patients. **(A)** illustrates the relationship between FT4 levels and MACEs, **(B)** delineates the relationship between the FT3/FT4 ratio and MACEs. Odds ratios are indicated by solid lines and 95% CIs by shaded areas.

In the subgroup analysis, we observed a significant association between FT4 levels and the FT3/FT4 ratio (per 1 SD increase) with the occurrence of MACEs in TAAAD patients. Then, we employed a RCS model to evaluate the potential non-linear relationships. The RCS model for FT4 (**Figure 3A**, nonlinearity P-value = 0.2) and the RCS model for the FT3/FT4 ratio (**Figure 3B**, nonlinearity P-value = 0.2) both indicated no significant nonlinear relationships.

### 3.5 Mediating roles of renal function and coagulation in MACEs

Logistic regression analyses have revealed a robust impact of FT4 and FT3/FT4 ratio on MACEs in TAAAD patients. Given these findings, we examined the mediating roles of biomarkers, including BUN, Cr, CysC, eGFR, PT, PTR, and PTINR, in the adverse effects of FT4 and the FT3/FT4 ratio on MACEs in TAAAD patients. **Table 5** demonstrates that BUN, Cr, CysC, eGFR, PTR, PT, and PTINR serve as partial mediators in the relationship between the FT3/FT4 ratio and the incidence of MACEs. Among these, CysC stands out with the most pronounced mediating proportion, constituting 30.5%. Conversely, FT4 predominantly mediates its effect by influencing coagulation function indicators.

**Table 5:** Mediating roles of FT3/FT4 and FT4 in MACEs occurrence in TAAAD.

Independent variable	Mediator	Total effect		Indirect effect		Direct effect		Proportion mediated, % (95% CI)
		Coefficient (95% CI)	*P*-value	Coefficient (95% CI)	*P*-value	Coefficient (95% CI)	*P*-value	
FT3/FT4	BUN	-0.45462 (-0.67875, -0.20556)	0.004	-0.06283 (-0.13636, -0.01296)	0.004	-0.39178 (-0.62886, -0.10874)	0.010	13.8 (2.7, 48.4)
	Cr	-0.46676 (-0.68142, -0.19870)	0.004	-0.07923 (-0.16305, -0.02601)	<0.001	-0.38753 (-0.61923, -0.08696)	0.014	17.0 (4.6, 56.1)
	CysC	-0.40355 (-0.69209, -0.02119)	0.040	-0.12324 (-0.23807, -0.03510)	0.004	-0.28031 (-0.60758, 0.12701)	0.168	30.5 (2.9, 192.8)
	eGFR	-0.47015 (-0.68231, -0.20240)	0.004	-0.03927 (-0.10311, -0.00345)	0.014	-0.43088 (-0.65045, -0.14728)	0.010	8.3 (0.5, 34.8)
	PTR	-0.40971 (-0.61808, -0.13312)	0.010	-0.07391 (-0.17455, -0.01168)	0.010	-0.33580 (-0.57196, -0.01983)	0.040	18.0 (2.1, 78.1)
	PT	-0.40978 (-0.61828, -0.13273)	0.010	-0.07234 (-0.17412, -0.01019)	0.012	-0.33744 (-0.57336, -0.02208)	0.040	17.7 (1.7, 77.5)
	PTINR	-0.40197 (-0.61789, -0.11467)	0.008	-0.07622 (-0.18442, -0.01598)	0.006	-0.32575 (-0.56608, -0.00480)	0.048	19.0 (3.3, 87.3)
FT4	BUN	0.01180 (0.00660, 0.01366)	0.002	0.00024 (-0.00073, 0.00137)	0.606	0.01156 (0.00661, 0.01342)	<0.001	2.0 (-7.5, 13.4)
	Cr	0.01220 (0.00582, 0.01359)	0.004	0.00088 (-0.00037, 0.00271)	0.206	0.01132 (0.00505, 0.01305)	0.004	7.2 (-3.3, 28.5)
	CysC	0.01097 (0.00016, 0.01366)	0.050	0.00187 (0.00002, 0.00450)	0.050	0.00910 (-0.00185, 0.01267)	0.076	17.0 (-10.7, 86.9)
	eGFR	0.01167 (0.00602, 0.01341)	0.004	0.00036 (-0.00061, 0	0.456	0.01132 (0.00536, 0.01317)	0.004	3.0 (-5.8, 18.3)
	PTR	0.01069 (0.00214, 0.01324)	0.022	0.00200 (0.00047, 0.00517)	0.006	0.00869 (-0.00089, 0.01175)	0.074	18.7 (3.1, 100.8)
	PT	0.01069 (0.00214, 0.01323)	0.022	0.00195 (0.00042, 0.00500)	0.008	0.00874 (-0.00086, 0.01177)	0.066	18.2 (2.8, 95.6)
	PTINR	0.01073 (0.00209, 0.01318)	0.026	0.00209 (0.00053, 0.00566)	0.002	0.00864 (-0.00141, 0.01163)	0.072	19.5 (3.4, 93.0)

Covariates in the models were age, gender, hypertension, diabetes, and surgical intervention.

BUN, Blood Urea Nitrogen; Cr, Creatinine; CysC, Cystatin C; PTR, Prothrombin Ratio; eGFR, Estimated Glomerular Filtration Rate; PT, Prothrombin Time; PTINR, Prothrombin Time International Normalized Ratio; MACEs, Major Adverse Cardiovascular Events; TAAAD, Type A Acute Aortic Dissection.

### 3.6 Multivariable mediation effects on the association between FT3/FT4 ratios and MACEs

**Figure 4:**
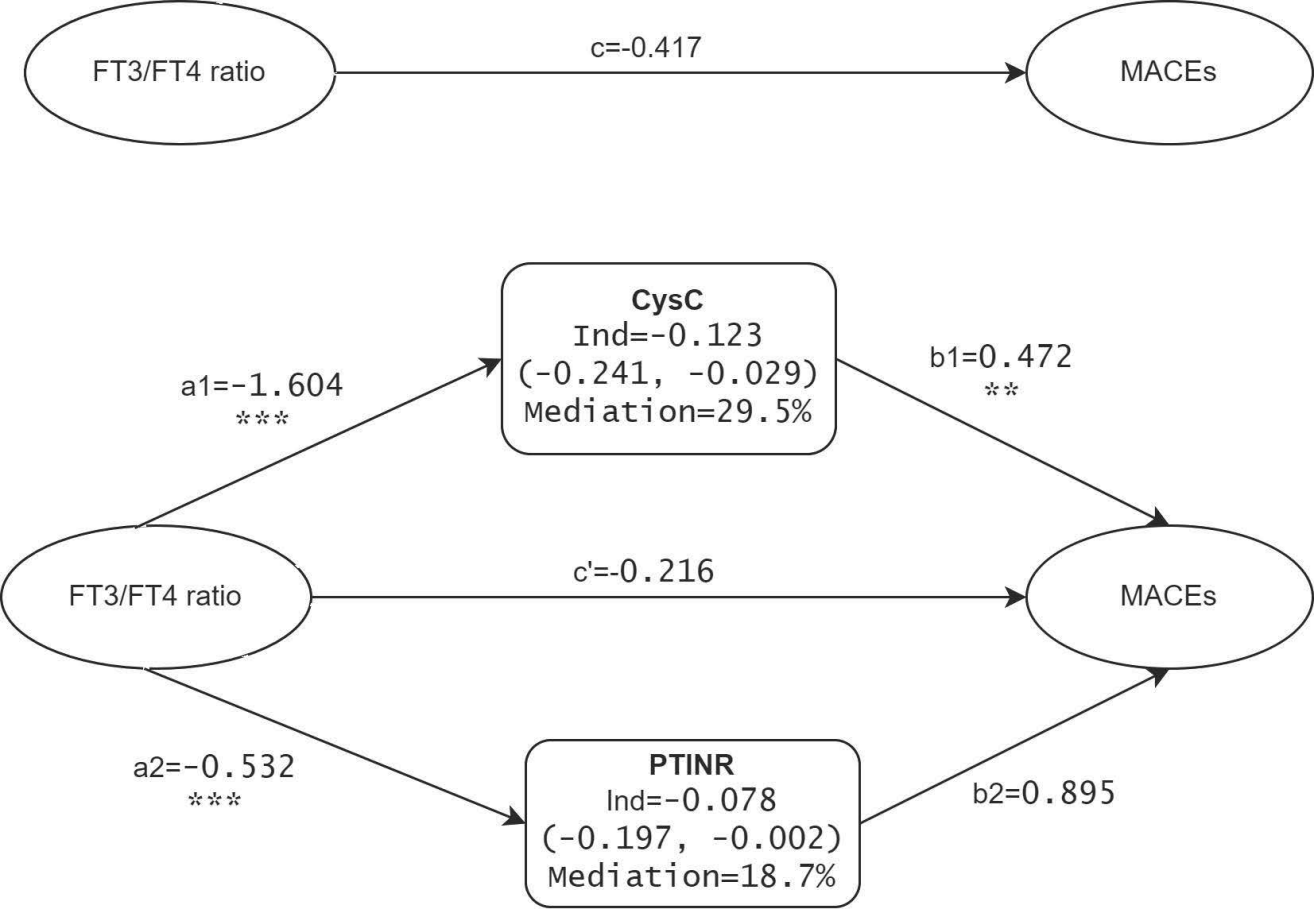
Mediation effects of CysC and PTINR on the FT3/FT4 ratio-MACEs association in TAAAD patients. a, b, c, and c’ are standardized regression coefficients; c=total effect; c’=direct effect; Ind=indirect effect. ** p <.01, *** p <.001.

The above results show that the FT3/FT4 ratio, through mediation by renal function markers and coagulation profiles, impacts the incidence of MACEs in TAAAD patients. To enhance the precision of these findings, we advanced to a multivariate parallel mediation model, incorporating CysC and PTINR—mediators with significant effect sizes in the initial analysis—to formulate a multiple mediator model without assuming inter-mediator interactions. **Figure 4** presents the mediation model parameters in TAAAD patients with the FT3/FT4 ratio as the predictor, CysC and PTINR as the mediators, and MACEs as the outcome, adjusting for sex, age, hypertension, diabetes, and surgery. The model corroborates the findings from the simple mediation analysis. The results substantiate the mediating roles of CysC and PTINR in the association between the FT3/FT4 ratio and MACEs, with a notably stronger mediating effect by CysC (mediating proportion 29.5%).

## 4 Discussion

The primary findings of our study indicate that FT4 levels and FT3/FT4 ratios are associated with MACEs and in-hospital mortality in AAD patients. Elevated FT4 levels increase the risk of MACEs and all-cause mortality, whereas a higher FT3/FT4 ratio is associated with reduced risk of MACEs. This association was particularly pronounced in TAAAD patients. Mediating analysis suggested that both renal function and coagulation acted as intermediaries in the relationship between the FT3/FT4 ratio and MACEs, with renal function playing a more significant role. Conversely, FT4 predominantly influenced MACEs indirectly through its impact on coagulation. These findings offer a new perspective on the role of thyroid function in determining the prognosis of aortic dissection patients.

Thyroid hormones exert direct effects on the cardiovascular system. They manifest direct genomic actions on cardiomyocytes, modulating the expression of target genes, and also exhibit non-genomic effects on ion channels within cardiomyocyte membranes (4). Even subtle variations in thyroid hormone concentrations can perturb cardiovascular physiology, leading to manifestations such as endothelial dysfunction, alterations in blood pressure, myocardial dysfunction, and lipid anomalies (3). Extensive literature underscores the nexus between thyroid hormones and progression of cardiovascular ailments, such as the independent predictive value of hypothyroidism or low T3 syndrome for cardiovascular adverse events and/or death in AMI patients (5, 32), the relationship between thyroid function and risk of atrial fibrillation and stroke (33), and the relationship between thyroid function and incidence and mortality of atherosclerotic cardiovascular disease (34).

Our study finds that high levels of FT4 and low FT3/FT4 ratios are associated with a high incidence of MACE in TAAAD patients. This association is likely due to reduced peripheral conversion of FT4 into FT3, suggesting the possible presence of low T3 syndrome in these patients. Low T3 syndrome reflects a state of non-thyroidal illness, where reduced levels of T3 are an adaptive response to acute illness but may also contribute to adverse outcomes (35, 36). It would be interesting to assess the role of low T3 syndrome in AAD patients, explore the underlying mechanisms, and consider its potential as a therapeutic target. Future studies should focus on these aspects to better understand and manage the condition.

We find that thyroid hormones can induce alterations in both renal function and coagulation. This is reflected by altered levels of CysC, BUN, Cr, and various coagulation parameters, such as PT and PTINR, ultimately impacting the prognosis of AAD patients. Such findings align with existing research. For instance, studies have reported the influence of FT3 levels on postoperative acute renal failure in aortic dissection patients (13), suggesting that thyroid function may play a role in AAD patient mortality through renal impairment.

The mechanisms behind this effect are multifaceted. Thyroid hormones are known to impact renal function through various mechanisms, including affecting renal blood flow, the RAS system in the kidneys, renal tubular ion transport, and glomerular filtration rate (37). Studies have found that higher FT4 levels are associated with risk of chronic kidney disease (CKD) and a rapid decline in eGFR (38). Conversely, higher FT3 levels are associated with a decrease in all-cause mortality and renal function indicators (39). A low FT3/FT4 ratio is associated with an increased mortality rate and worsening prognosis in CKD patients (40), and this ratio also associates with the risk of diabetic nephropathy (41). These renal abnormalities can severely impact AAD patients, increasing risks of in-hospital mortality, stroke, and renal failure (42, 43).

Furthermore, both *in vitro* and clinical studies confirm that thyroid hormones, particularly free hormones such as FT4, significantly influence coagulation functions. Elevated levels of FT4 are associated with an augmented risk of thrombosis, indicative of a heightened likelihood of thromboembolic diseases (44–46), and thyroid hormones regulate coagulation processes through various mechanisms. For instance, 3,5,3’-triiodothyronine (T3) interacts with nuclear thyroid hormone receptors, affecting the synthesis and secretion of coagulation factors, vascular endothelial functions, platelet activity, and the fibrinolytic system (47). Beyond genomic actions, recent findings have highlighted that nongenomic mechanisms, initiated at the L-thyroxine (T4) receptor on platelet integrin αvβ3, also exhibit prothrombotic properties. These T4-induced mechanisms entail platelet activation and the production of cytokines and chemokines, such as CX3CL1, with procoagulant activities. Abnormal coagulation further elevates the risk of adverse outcomes in AAD patients (20, 48).

While it is theoretically plausible that thyroid hormones could affect AAD patient prognosis, literature on this topic is sparse. The phenomenon of ‘over-adjustment’ in statistical models (49), where mediating variables might dilute or obscure the effect of the independent variable, is one possible factor among others that could contribute to the limited reports in the literature. Thus, when analyzing thyroid hormone impact on AAD prognosis, it is crucial to avoid including these mediating variables as corrective covariates.

Although our study provides insightful findings, it has some limitations. Firstly, due to its observational nature, we cannot assert causality between thyroid hormone levels and clinical outcomes in AAD patients. Longitudinal studies or randomized controlled trials would be more definitive in this regard. Secondly, our analysis was based on single-time measurements of thyroid hormone levels, renal function, and coagulation. Future studies might benefit from multiple measurements over time to account for potential fluctuations in these parameters. Thirdly, although we adjusted for a wide range of confounding factors, there might be other unmeasured variables that could have influenced our results. For instance, vascular calcification and atherosclerosis have been reported as independent risk factors for MACEs in aneurysm patients (50–54); the presence of a bicuspid aortic valve (55), a family history of aortic dissection or other aortic pathologies (56), and patient compliance data (57) could also influence prognosis. However, our study did not collect data on these factors. Future research should consider incorporating these factors to better understand their impact on the prognosis of AAD patients. Lastly, the findings from our single-center study may not be generalizable to other settings. Multicenter studies would help to validate our results and enhance their applicability to a broader population.

Nevertheless, our findings hold significant theoretical and practical value. They propose potential pathways through which thyroid hormones may influence AAD prognosis via renal and coagulation functions. This insight not only enhances our understanding of aortic dissection prognosis, but also suggests new biological mechanisms, aiding in risk stratification and personalized treatment for AAD patients. However, it is imperative to note that while our findings indicate a significant association, causative relationships should be interpreted with caution given the inherent limitations of observational research.

## 5 Conclusion

FT4 and the FT3/FT4 ratio are independent predictors of MACEs in AAD patients. The findings are especially relevant for TAAAD patients. Renal and coagulation functions serve as mediators in these associations. Our results underscore the importance of considering the FT3/FT4 ratio alongside FT4 in the prognostic evaluation of AAD patients.

## Data availability statement

The raw data supporting the conclusions of this article will be made available by the authors, without undue reservation.

## Ethics statement

The studies involving humans were approved by the Ethics Committee of the First Affiliated Hospital of Shantou University Medical College (B-2022-207). The studies were conducted in accordance with the local legislation and institutional requirements. The participants provided their written informed consent to participate in this study.

## Author contributions

XS: Formal analysis, Funding acquisition, Investigation, Methodology, Visualization, Writing – original draft, Writing – review & editing. SW: Data curation, Funding acquisition, Investigation, Methodology, Writing – original draft. JY: Conceptualization, Data curation, Formal analysis, Funding acquisition, Writing – original draft. HY: Formal analysis, Investigation, Validation, Writing – original draft. SZ: Investigation, Methodology, Writing – original draft. HW: Investigation, Methodology, Writing – original draft. SY: Conceptualization, Methodology, Resources, Supervision, Validation, Writing – original draft, Writing – review & editing. WL: Conceptualization, Methodology, Project administration, Resources, Supervision, Writing – review & editing.
